# Prognostic values of apoptosis-stimulating P53-binding protein 1 and 2 and their relationships with clinical characteristics of esophageal squamous cell carcinoma patients: a retrospective study

**DOI:** 10.1186/s40880-016-0169-0

**Published:** 2017-01-19

**Authors:** Xiao-Feng Xie, Qing Yang, Jun Chi, Xian-Zi Yang, Hui-Yun Wang, Guo-Liang Xu

**Affiliations:** 1 State Key Laboratory of Oncology in South China, Sun Yat-sen University Cancer Center, 651 Dongfeng East Road, Guangzhou, P. R. China; 2 Department of Endoscopy and Laser, Sun Yat-sen University Cancer Center, 651 Dongfeng East Road, Guangzhou, Guangdong 510060 P. R. China; 3 Guangdong Esophageal Cancer Institute, Guangzhou, Guangdong 510060 P. R. China

**Keywords:** Apoptosis-stimulating protein of P53 1 and 2, P53, Prognosis, Esophageal squamous cell carcinoma

## Abstract

**Background:**

Esophageal squamous cell carcinoma (ESCC) is a leading cause of cancer-related death, and new prognostic biomarkers are urgently needed. Apoptosis-stimulating P53-binding protein 1 (ASPP1) and 2 (ASPP2) have been reported to play important roles in the development, progression, metastasis, and prognosis of cancers, but their roles in ESCC have not been elucidated. In this study, we examined the expression of ASPP1 and ASPP2 in ESCC to evaluate their prognostic values.

**Methods:**

The protein expression of ASPP1, ASPP2, and P53 in 175 specimens of ESCC was detected using immunohistochemical staining; their expression in cancerous and noncancerous tissues was scored according to the staining intensity and the percentage of stained cells. The associations of ASPP1, ASPP2, and P53 with clinicopathologic parameters, overall survival (OS), and disease-free survival (DFS) were analyzed.

**Results:**

The protein expression levels of ASPP2 and P53 were significantly higher in cancerous tissues than in paired noncancerous tissues (*P* < 0.001), whereas the expression levels of ASPP1 in the two groups were similar. In ESCCs, ASPP1 expression was significantly associated with histological differentiation (*P* = 0.002) and invasive depth (*P* = 0.014); ASPP2 expression was associated with age (*P* = 0.029) and histological differentiation (*P* < 0.001); and P53 expression was associated with age (*P* = 0.021) and tumor size (*P* = 0.040). No correlations were found between ASPP1, ASPP2, and P53 expression. Survival analysis revealed that high ASPP2 expression was significantly associated with increased 5-year OS (*P* = 0.001) and DFS rates (*P* = 0.010) and that high P53 expression was significantly associated with a reduced 5-year DFS rate of ESCC patients (*P* = 0.015). Multivariate Cox analysis indicated that ASPP2 was an independent predictor of OS [hazard ratio (HR): 0.541, 95% confidence interval (CI) 0.363–0.804] and DFS (HR: 0.599, 95% CI 0.404–0.888) of ESCC patients and that P53 was an independent predictor of DFS (HR: 2.161, 95% CI 1.100–4.245).

**Conclusions:**

ASPP1 might be involved in the progression of ESCC, and ASPP2 was a potential prognostic biomarker of ESCC and should be evaluated in future studies.

## Background

Esophageal carcinoma is the eighth most prevalent cancer and the seventh leading cause of cancer-related death worldwide [1] and is the fourth leading cause of cancer-related death in China [2]. The most common histological type of esophageal carcinoma in China is esophageal squamous cell carcinoma (ESCC) [1]. The 5-year overall survival (OS) rate of ESCC patients after esophagectomy has significantly increased over the past several decades but remains approximately 20% [3–5]. The late appearance of symptoms, rapid progression, local recurrence, and metastasis are the main reasons for the low survival rate [6–9]. However, the molecular mechanism of ESCC genesis and progression is not well understood, and biomarkers for predicting clinical outcome of ESCC patients are unavailable. Therefore, there is an urgent need to identify sensitive and specific biomarkers for ESCC.

The well-known tumor suppressor protein P53 has been reported to be involved in the development of many types of cancer, including esophageal carcinoma [10]. Over 10 years ago, two proteins both containing a large number of ankyrin (Ank) repeat sequences, an Src-homology 3 (SH3) domain, and a proline-rich domain were identified as activators for P53 apoptotic function and were thus named apoptosis-stimulating P53-binding protein 1 (ASPP1) and 2 (ASPP2) [11, 12]. ASPP1 and ASPP2 have been reported to play important roles in the developement, progression, metastasis, and prognosis of cancer. Samuels-Lev et al. [11] and Slee et al. [12] have demonstrated a specific effect of ASPP1 and ASPP2 in breast cancer on the apoptotic and transactivation functions of P53 for the expression of proapoptotic targets, such as Bax, P53 up-regulated modulator of apoptosis (PUMA), and P53-induced gene 3 (PIG3), through combination with P53 via the Ank sequence and SH3 domain. However, ASPP1 and ASPP2 failed to affect the cell cycle arrest function of P53 under the same conditions. ASPP1 and ASPP2 could mediate the cell fate transition between life and death. Sgroi et al. [13] found that invasive and metastatic breast cancer tissue exhibited reduced ASPP2 expression compared with normal breast tissue, suggesting a possible role of ASPP2 in breast cancer progression and metastasis. Mak et al. [14] reported the down-regulation of ASPP2 in choriocarcinoma, which contributed to the increased migratory potential of the tumor cells. High ASPP2 expression has been reported to predict good prognosis in some tumors, including breast cancer [15], non-small cell lung cancer [16], diffuse large B cell lymphoma, and follicular center lymphoma [17]. Moreover, a recent study showed that ASPP2 also induced apoptosis through a P53-independent mechanism [18]. Additionally, ASPP2 plays an important role in regulating the nuclear factor kappa B subunit 1 (NFκB1) inflammatory pathway [19] and in modulating autophagy [18, 20] and cell polarity [21, 22].

Although ASPP1 and ASPP2 have been reported to play crucial roles in the development of several malignancies, their involvement in ESCC development remains undetermined. In this study, we detected the expression of ASPP1, ASPP2, and P53 in ESCCs using immunohistochemistry to investigate their prognostic values and relationships with clinical characteristics of ESCC patients.

## Methods

### Patient selection

ESCC patients who underwent curative esophagectomy between November 2004 and December 2006 at Sun Yat-sen University Cancer Center (SYSUCC, Guangzhou, China) were selected for this study. All procedures involving human participants were in accordance with the standards of the Ethics Committee of SYSUCC. Informed consent was obtained from all patients included in the study. The following criteria were used for patient selection: (1) no radiotherapy, chemotherapy, or other cancer treatment before surgery; (2) ESCC diagnosed with pathologic examination; (3) R0 resection confirmed with postoperative pathologic examination; and (4) no distant metastasis diagnosed with imaging before surgery, including supraclavicular and abdominal para-aortic lymph node metastasis. Patients were ineligible if they had cervical ESCC, stage T4b ESCC at the time of diagnosis, other concomitant malignancies, or severe organ disorders. Cancers were pathologically restaged according to the 7th edition of the Union for International Cancer Control (UICC) tumor-node-metastasis (TNM) staging system.

### Follow-up

All patients were followed up from operation time to December 31, 2016 by telephone and return visits, with an interval of 3 months, and death was considered an event. Post-operative metastasis and recurrence were diagnosed on the basis of clinical examination, imaging assessment (e.g., barium meal, computed tomography, positron emission tomography-computed tomography, and endoscopic examination), and operative and pathologic examination. OS was calculated from the time of treatment to the date of death or the last follow-up. Disease-free survival (DFS) was defined as the duration between operation and relapse, metastasis, or cancer-related diseases. Clinicopathologic data were obtained from pathologic reports, medical records, laboratory examination, and imaging, primarily including information of age, gender, histological differentiation, tumor location, pTNM stage, T status (invasive depth), N status (lymph node metastasis), tumor size, smoking habit, alcohol consumption, postoperative radiotherapy and chemotherapy.

### Immunohistochemistry

The appropriate paraffin-embedded specimen blocks of each case of ESCC were obtained from the Department of Pathology. If possible, adjacent non-cancerous tissue specimens were processed and compared with the cancerous specimens as matched pairs. Tissue sections (4 µm thick) were dried at 60°C for 3–4 h, deparaffinized with three 10-min washes in xylene, and rehydrated in decreasing concentrations of ethanol in distilled water. Then, the tissue sections were treated with 3% hydrogen peroxide for 10 min to block endogenous peroxidase activity. After washing 10 min in phosphate buffer solution (PBS, ZSGB-BIO, Beijing, China) for three times, the sections were soaked in boiling sodium citrate buffer (ZSGB-BIO) for 20 min in microwave oven. When cooled to room temperature, the sections were washed 5 min in PBS for three times and were incubated overnight at 4°C with rabbit polyclonal anti-ASPP1 antibody (bs-1282R; dilution, 1:100; Bioss, Beijing, China), rabbit polyclonal anti-ASPP2 antibody (bs-1283R; dilution, 1:300; Bioss), or mouse monoclonal antibody against wild-type and mutated P53 (ZM-0408; dilution, 1:100; ZSGB-BIO) and followed by incubation with reagent A [secondary antibody of GTVision™ III Detection System/Mo&Rb, Gene Tech Biotechnology (Shanghai) Company Limited, Shanghai, China] for 30 min at 37°C. The colorimetric reaction was observed under a microscope after incubating with reagent B (GTVision™ III Detection System/Mo&Rb). Sections were counterstained with 5% hematoxylin for 5 min. Finally, the sections were blued in 1% hydrochloric-acid alcohol, dehydrated in increasing concentrations of ethanol, cleared with xylene, and mounted in neutral gum under a coverslip. The sections treated without primary antibody were used as negative control. For positive controls, non-small cell lung cancer (for ASPP1 and ASPP2) [16] and ESCC sections (for P53) [23] were stained using the same protocol.

### Evaluation of immunohistochemical staining

Using a high-power (400×) microscope (Eclipse 80i, Nikon, Tokyo, Japan), ASPP1, ASPP2, and P53 expression was evaluated by two experienced pathologists independently, without knowledge of the clinical information. For each section, five random non-overlapping fields containing at least 200 cells per field were observed and scored based on the percentage of positively stained cells (0%–100%) and the staining intensity (score 0 for negative staining, 1 for weak staining, 2 for moderate staining, and 3 for strong staining). The immunoreactive score (IRS) was calculated with the formula, the percentage of positive cells × the staining intensity × 100, to produce a value between 0 and 300. Patients were divided into low and high expression groups according to the median IRSs.

### Statistical analysis

All data were analyzed using SPSS16.0 statistical software (SPSS Inc., Chicago, IL, USA) and GraphPad Prism 5 software (GraphPad Software Inc., La Jolla, CA, USA). Paired and unpaired Student’s *t* tests were performed to compare ASPP1, ASPP2, and P53 expression between ESCCs and noncancerous tissues. The relationships of ASPP1, ASPP2, and P53 expression with the clinicopathologic parameters were assessed using Chi-square and Fisher’s exact tests. Spearman’s rank correlation was used to analyze the correlations between ASPP1, ASPP2, and P53 expression. Kaplan–Meier survival analysis and log-rank tests were used to evaluate the 5-year OS and DFS rates of ESCC patients. Cox univariate analysis was used to determine the prognostic significance of variables, and Cox multivariate analysis was applied to identify independent prognostic factors for ESCC. For all results, *P* < 0.05 was considered statistically significant.

## Results

### The expression of ASPP1, ASPP2, and P53 in ESCCs and noncancerous tissues

We examined the expression of ASPP1, ASPP2, and P53 in 175 ESCC tissues and 105 paired noncancerous tissues using immunohistochemical staining. The cases with insufficient tissue for immunohistochemical staining were excluded from relevant analysis. Patients’ characteristics are listed in Table 1. The median IRSs were 100 for ASPP1, 110 for ASPP2, and 100 for P53.

**Table 1:** Associations between the expression of apoptosis-stimulating P53-binding protein 1 (ASPP1) and 2 (ASPP2) and P53 and clinicopathologic characteristics of the 175 esophageal squamous cell carcinoma (ESCC) patients

Characteristic	ASPP1 expression [cases (%)]		*P* value	ASPP2 expression [cases (%)]		*P* value	P53 expression [cases (%)]		*P* value
	Low	High		Low	High		Low	High	
Total^a^	79	95		75	98		82	91	
Gender			0.569			0.713			0.794
Male	56 (70.9)	71 (74.7)		54 (72.0)	73 (74.5)		58 (70.7)	66 (72.5)	
Female	23 (29.1)	24 (25.3)		21 (28.0)	25 (25.5)		24 (29.3)	25 (27.5)	
Age (years)			0.495			0.029			0.021
≤60	57 (72.2)	64 (67.4)		45 (60.0)	74 (75.5)		63 (76.8)	55 (60.4)	
>60	22 (27.8)	31 (32.6)		30 (40.0)	24 (24.5)		19 (23.2)	36 (39.6)	
Histological differentiation			0.002			<0.001			0.301
Well	14 (17.7)	40 (42.1)		15 (20.0)	39 (39.8)		21 (25.6)	32 (35.2)	
Moderate	48 (60.8)	41 (43.2)		38 (50.7)	51 (52.0)		44 (53.7)	46 (50.5)	
Poor	17 (21.5)	14 (14.7)		22 (29.3)	8 (8.2)		17 (20.7)	13 (14.3)	
Tumor location			0.459			0.311			0.446
Upper thoracic esophagus	8 (10.1)	5 (5.2)		7 (9.3)	7 (7.1)		7 (8.5)	7 (7.7)	
Middle thoracic esophagus	55 (69.6)	68 (71.6)		47 (62.7)	72 (73.5)		60 (73.2)	60 (65.9)	
Lower thoracic esophagus	16 (20.3)	22 (23.2)		21 (28.0)	19 (19.4)		15 (18.3)	24 (26.4)	
pTNM stage			0.628			0.148			0.400
I–II	37 (46.8)	48 (50.5)		33 (44.0)	54 (55.1)		44 (53.7)	43 (47.3)	
III	42 (53.2)	47 (49.5)		42 (56.0)	44 (44.9)		38 (46.3)	48 (52.7)	
Invasive depth			0.014			0.920			0.145
T1–2	14 (17.7)	30 (31.6)		18 (24.0)	26 (26.5)		26 (31.7)	18 (19.8)	
T3	62 (78.5)	55 (57.9)		51 (68.0)	65 (66.3)		49 (59.8)	67 (73.6)	
T4	3 (3.8)	10 (10.5)		6 (8.0)	7 (7.2)		7 (8.5)	6 (6.6)	
Lymph node metastasis			0.708			0.162			0.156
Absence	36 (45.6)	46 (48.4)		31 (41.3)	51 (52.0)		44 (53.7)	39 (42.9)	
Presence	43 (54.4)	49 (51.6)		44 (58.7)	47 (48.0)		38 (46.3)	52 (57.1)	
Tumor size (cm)			0.423			0.463			0.040
<5	61 (77.2)	78 (82.1)		57 (76.0)	79 (80.6)		70 (85.4)	66 (72.5)	
≥5	18 (22.8)	17 (17.9)		18 (24.0)	19 (19.4)		12 (14.6)	25 (27.5)	
Smoking			0.924			0.807			0.171
Yes	46 (58.2)	56 (58.9)		43 (57.3)	58 (59.2)		42 (51.2)	56 (61.5)	
No	33 (41.8)	39 (41.1)		32 (42.7)	40 (40.8)		40 (48.8)	35 (38.5)	
Alcohol drinking			0.537			0.478			0.568
Yes	25 (31.6)	26 (27.4)		20 (26.7)	31 (31.6)		22 (26.8)	28 (30.8)	
No	54 (68.4)	69 (72.6)		55 (73.3)	67 (68.4)		60 (73.2)	63 (69.2)	

^a^The cases with insufficient tissue for immunohistochemical staining were excluded from relevant analysis

**Fig. 1:**
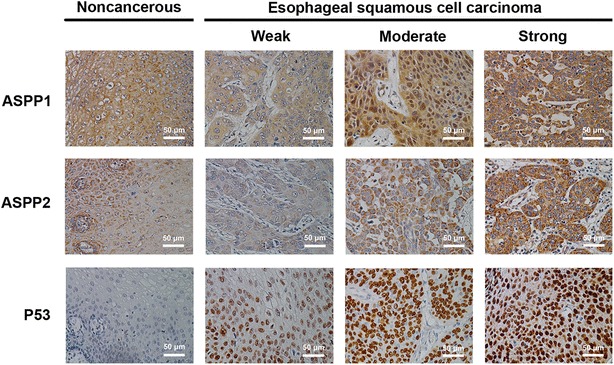
Immunohistochemical staining of apoptosis-stimulating P53-binding protein 1 (ASPP1) and 2 (ASPP2) and P53 in esophageal squamous cell carcinoma (ESCC) and noncancerous tissues. ASPP1 is primarily expressed in the cytoplasm and is strongly positive in noncancerous tissues and ESCCs. ASPP2 is primarily expressed in the perinuclear region and is moderately positive in noncancerous tissues while strongly positive in ESCC. P53 is primarily expressed in the nucleus in most ESCCs, but is seldom detected in noncancerous tissues

**Fig. 2:**
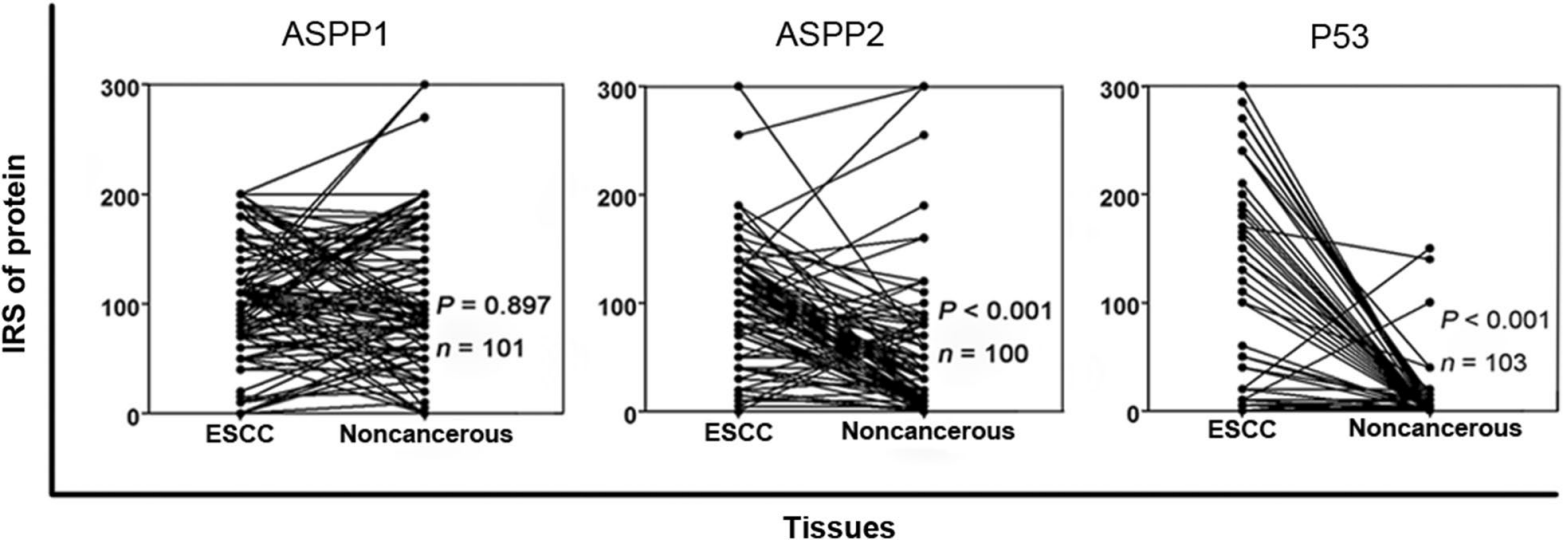
Expression of ASPP1, ASPP2, and P53 in paired ESCC and noncancerous tissues. IRS: immunoreactive score. The cases with insufficient tissue for immunohistochemical staining were excluded from relevant analysis. ASPP1 expression is not significantly different between the paired ESCC and noncancerous tissues (*P* = 0.897). Both ASPP2 and P53 expression are significantly higher in ESCCs than in noncancerous tissues (both *P* < 0.001)

**Fig. 3:**
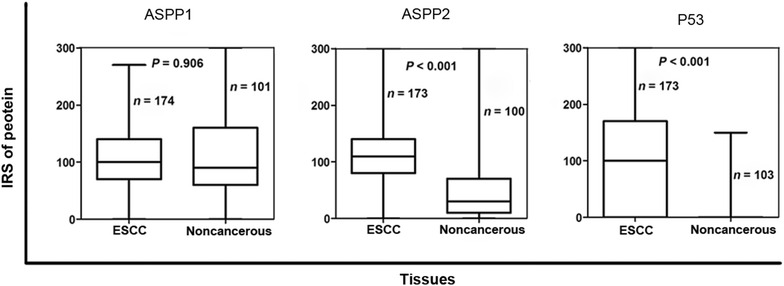
Average levels of ASPP1, ASPP2, and P53 expression in unpaired ESCC and noncancerous tissues. The cases with insufficient tissue for immunohistochemical staining were excluded from relevant analysis. Average level of ASPP1 expression is not significantly different between ESCC and noncancerous tissues (*P* = 0.904). Average levels of both ASPP2 and P53 expression are significantly higher in ESCCs than in noncancerous tissues (both *P* < 0.001). The *horizontal lines* in the boxes represent the average expression levels

Immunohistochemical staining showed that ASPP1 was expressed in the cytoplasm, ASPP2 was expressed primarily in the perinuclear cytoplasm, and P53 was expressed in the nucleus (Fig. 1). In the paired analysis, the rate of high ASPP1 expression was similar between ESCCs and noncancerous tissues [44.6% (45/101) vs. 45.5% (46/101), *P* = 0.897], whereas the rates of high ASPP2 and P53 expression were both significantly higher in ESCCs than in noncancerous tissues [51.0% (51/100) vs. 10.0% (10/100), *P* < 0.001; 54.4% (56/103) vs. 2.9% (3/103), *P* < 0.001] (Fig. 2). The results of the unpaired analysis were consistent with those of the paired analysis (Fig. 3).

### Relationships between ASPP1, ASPP2, P53 expression and clinical characteristics of ESCC patients

To better understand the clinical significance of ASPP1, ASPP2, and P53 in ESCC, we examined the relationships between their expression and clinical characteristics of ESCC patients (Table 1). ASPP1 expression was associated with histological differentiation (*P* = 0.002) and invasive depth (*P* = 0.014). ASPP2 expression was associated with histological differentiation (*P* < 0.001) and age (*P* = 0.029). P53 expression was associated with tumor size (*P* = 0.040) and age (*P* = 0.021). However, no correlation was found between the expression of ASPP1 and P53 (*R* = 0.033, *P* = 0.671), between the expression of ASPP2 and P53 (*R* = 0.036, *P* = 0.647), or between the expression of ASPP1 and ASPP2 (*R* = 0.045, *P* = 0.895).

### Relationships between ASPP1, ASPP2, P53 expression and survival of ESCC patients

**Fig. 4:**
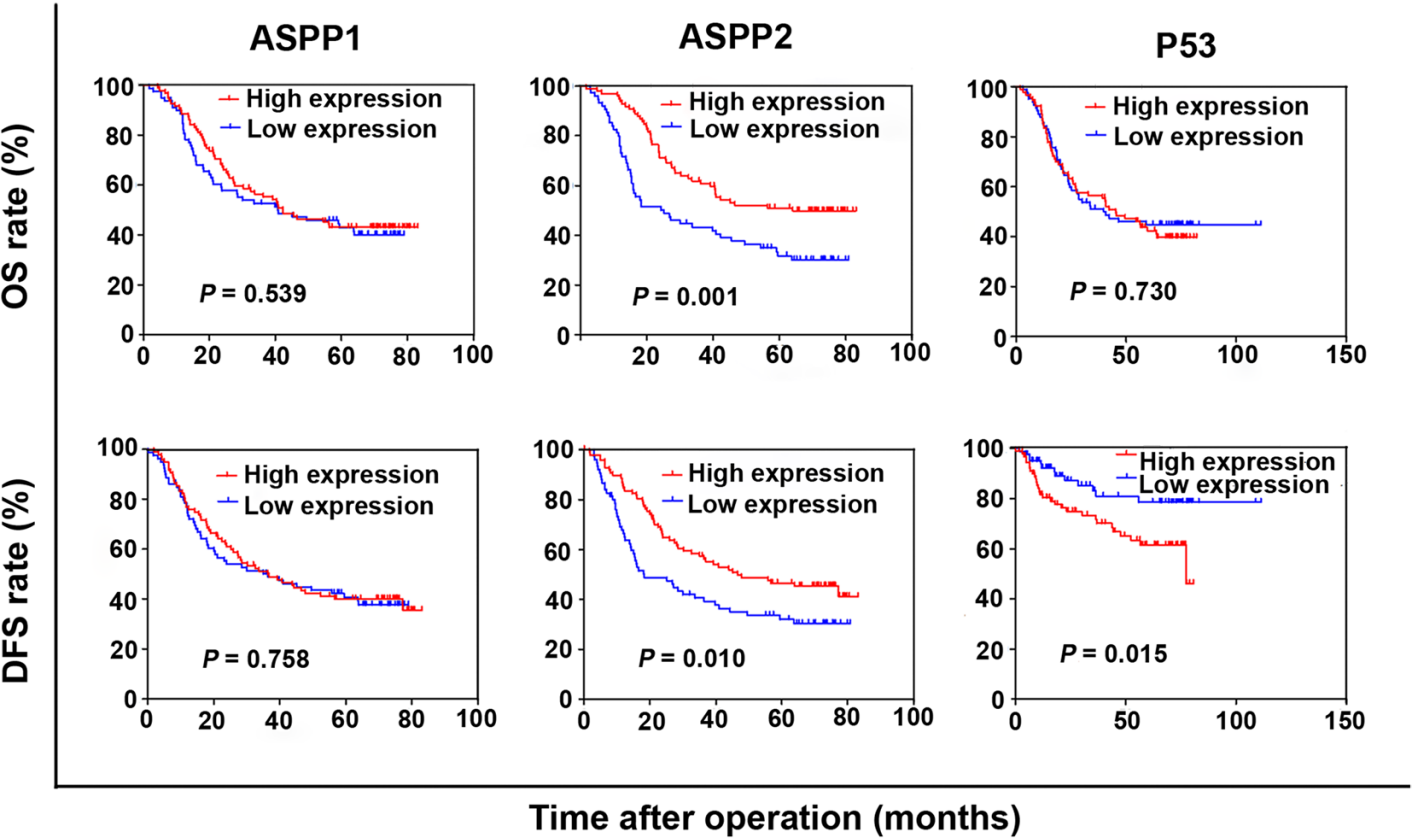
Kaplan–Meier survival curves of ESCC patients with low and high expression of ASPP1, ASPP2, and P53. The overall survival (OS) and disease-free survival (DFS) curves of patients with high ASPP1 expression are not significantly different from those of patients with low ASPP1 expression. Patients with high ASPP2 expression have higher 5-year OS and DFS rates than those with low ASPP2 expression. Patients with high P53 expression have a lower 5-year DFS rate than those with low P53 expression, whereas the 5-year OS rates were similar for both groups

To further evaluate the prognostic value of ASPP1, ASPP2, and P53 in ESCC, we performed Kaplan–Meier survival analysis and log-rank tests. ASPP1 expression was not associated with survival rate, whereas high ASPP2 expression was significantly associated with increased 5-year OS rate (51.0% vs. 31.7%, *P* = 0.001) and 5-year DFS rate as compared with low ASPP2 expression (46.5% vs. 32.0%, *P* = 0.010), and low P53 expression was associated with increased 5-year DFS rate as compared with high P53 expression (78.6% vs. 61.4%, *P* = 0.015) (Fig. 4).

Univariate Cox analysis also indicated that low ASPP2 expression was a significant predictor of short OS [hazard ratio (HR): 0.527, 95% confidence interval (CI) 0.355–0.782, *P* = 0.001] and DFS (HR: 0.606, 95% CI 0.412–0.891, *P* = 0.011) and that high P53 expression was a predictor of short DFS (HR: 2.198, 95% CI 1.144–4.224, *P* = 0.018); ASPP1 expression had no significant association with survival (Table 2). Multivariate Cox analysis indicated that low ASPP2 expression was an independent predictor of short OS (HR: 0.541, 95% CI 0.363–0.804, *P* = 0.002) and DFS (HR: 0.599, 95% CI 0.404–0.888, *P* = 0.011) and that low P53 expression was an independent predictor of long DFS (HR: 2.161, 95% CI 1.100–4.245, *P* = 0.025) (Table 3). Other significant predictors of OS and DFS included pTNM stage, invasive depth, lymph node metastasis, radiotherapy, and chemotherapy (Table 2), whereas only invasive depth, lymph node metastasis, and radiotherapy were independent predictors (Table 3).

**Table 2:** Univariate Cox regression analysis of predictors for OS and DFS of ESCC patients

Variable	OS			DFS		
	HR	95% CI	*P* value	HR	95% CI	*P* value
Gender (male vs. female)	1.367	0.893–2.093	0.150	1.343	0.881–2.046	0.170
Age (≤60 vs. >60 years)	1.168	0.769–1.774	0.466	1.040	0.688–1.572	0.852
Histological differentiation (well vs. moderate vs. poor)	1.192	0.895–1.587	0.230	1.123	0.848–1.487	0.419
Tumor location (upper vs. middle vs. lower thoracic esophagus)	0.935	0.642–1.363	0.728	0.980	0.676–1.420	0.915
pTNM stage (III vs. I–II)	2.401	1.594–3.616	<0.001	2.405	1.611–3.591	<0.001
Invasive depth (T4 vs. T3 vs. T1–2)	1.672	1.145–2.442	0.008	1.663	1.143–2.418	0.008
N status (N1-3 vs. N0)	2.443	1.609–3.708	<0.001	2.383	1.586–3.581	<0.001
Tumor size (<5 vs. ≥5 cm)	1.061	0.655–1.718	0.810	1.083	0.676–1.735	0.740
ASPP1 expression (low vs. high)	0.942	0.641–1.383	0.761	0.942	0.641–1.383	0.761
ASPP2 expression (high vs. low)	0.527	0.355–0.782	0.001	0.606	0.412–0.891	0.011
P53 expression (high vs. low)	1.072	0.721–1.596	0.731	2.198	1.144–4.224	0.018
Smoking (yes vs. no)	1.023	0.686–1.527	0.910	1.020	0.689–1.509	0.921
Alcohol drinking (yes vs. no)	0.869	0.568–1.329	0.517	0.786	0.521–1.185	0.251
Radiotherapy (yes vs. no)	0.742	0.421–1.307	0.301	0.567	0.333–0.967	0.037
Chemotherapy (yes vs. no)	0.681	0.432–1.072	0.097	0.580	0.374–0.900	0.015

*OS* overall survival, *DFS* disease-free survival, *HR* hazard ratio, *CI* confidence interval

**Table 3:** Multivariate Cox regression analysis of predictors for OS and DFS of ESCC patients

Variable	OS			DFS		
	HR	95% CI	*P* value	HR	95% CI	*P* value
Invasive depth (T4 vs. T3 vs. T1–2)	1.646	1.083–2.501	0.020	1.532	1.028–2.283	0.036
N status (N1–3 vs. N0)	2.248	1.474–3.426	<0.001	2.139	1.405–3.256	<0.001
ASPP2 expression (high vs. low)	0.541	0.363–0.804	0.002	0.599	0.404–0.888	0.011
P53 expression (high vs. low)	–	–	–	2.161	1.100–4.245	0.025
Radiotherapy (yes vs. no)	–	–	–	0.575	0.374–0.990	0.046

– The variables with a *P* value > 0.05 were not included in the multivariate analysis. Abbreviations as in Table 2

**Fig. 5:**
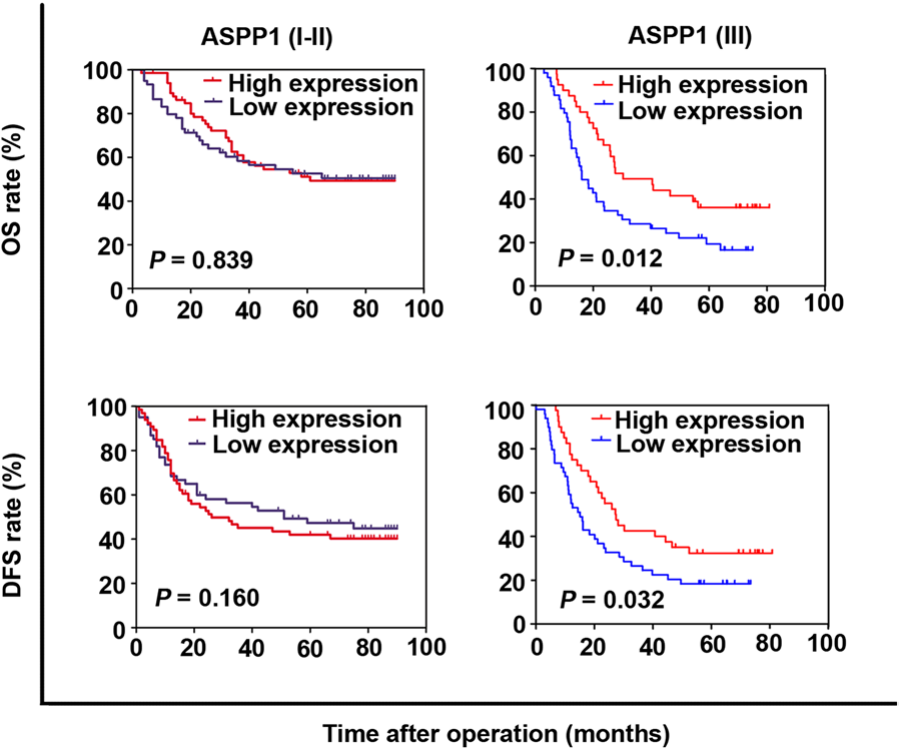
Kaplan–Meier survival curves of ESCC patients at different stages with low and high expression of ASPP1. For patients with stage I–II ESCC, the OS and DFS curves of patients with high ASPP1 expression are not significantly different from those of patients with low ASPP1 expression. High ASPP1 expression was associated with long OS and DFS in patients with stage III ESCC

Because tumor stage was closely associated with the prognosis of ESCC patient, we performed subgroup analysis according to the stages. For patients with stage I–II ESCC, the expression of ASPP1 showed no prognostic value; for patients with stage III ESCC, high ASPP1 expression was associated with long OS (*P* = 0.012) and DFS (*P* = 0.032) (Fig. 5). However, ASPP2 and P53 expression showed no prognostic values for patients with either stage I–II or III ESCC (data not shown).

## Discussion

In the present study, we demonstrated that ASPP2 expression was significantly increased in ESCC and was associated with histological differentiation and age. ASPP2 was an independent predictor of OS and DFS of ESCC patients. Although the expression of ASPP1 was not associated with overall prognosis, it was associated with the prognosis of patients with stage III disease.

ASPP2 is commonly considered a tumor suppressor, and its expression is often reduced in malignant tumors [14, 24, 25]. However, inconsistent with previous reports, our results indicated that ASPP2 expression was significantly increased in ESCCs as compared with noncancerous tissues, suggesting that ASPP2 might be involved in ESCC tumorigenesis. Interestingly, the expression of the well-known tumor suppressor *p53* was also reported to be increased in several types of cancer, including ESCC, and its high expression was related with a mutation [26, 27]. It is well known that protein phosphorylation and dephosphorylation serve as “signal switch” and are the most important mechanisms of signaling pathway regulation. Wild-type (unphosphorylated) P53 is usually undetectable using immunohistochemistry, whereas mutant (phosphorylated) P53 with dominant-negative activities and oncogenic properties can be detected because its half-life is much longer [28–32]. High expression of mutant (phosphorylated) P53 has been observed in many malignancies [11, 29, 33–35]. Protein expression is highly cell environment-specific and is regulated both genetically and epigenetically. Similar to P53, ASPP2 is also a tumor suppressor that is up-regulated in ESCC. We hypothesized that high ASPP2 expression in ESCC may be due to epigenetic alterations, such as phosphorylation and methylation.

Moreover, some proteins, such as nuclear factor erythroid-2 related factor 2 (NRF2), play important roles in protecting normal cells from tumorigenesis or disease development, but can be hijacked by cancer cells upon neoplasm initiation [36, 37]. It has been reported that NFR2 functions as a suppressor in carcinomas through autophagy-related [38] and inflammation-related cancer signaling pathways [39, 40]. Mounting evidence has suggested that ASPP2 also plays an antitumor role in malignancies through autophagy- [18] and inflammatory-related pathways independent of P53 [19]. Thus, we suggest that ASPP2 may function through similar pathways to NRF2. However, the underlying mechanism requires further investigation.

Furthermore, our results showed that high ASPP2 expression was associated with high degree of differentiation of ESCC, suggesting that ASPP2 may repress the progression of ESCC. We also found that high P53 expression was associated with large tumor size, indicating that mutant P53 expression may be involved in tumor progression, which is consistent with a recent study [23]. Although we did not detect a difference in ASPP1 expression between ESCCs and noncancerous tissues, high ASPP1 expression was associated with high degree of histological differentiation and shallow invasion of ESCCs, suggesting that high ASPP1 expression may inhibit tumor progression.

According to our results, low ASPP2 expression independently predicted poor prognosis of ESCC patients, which is consistent with previous reports of other cancers [15–17]. More aggressive therapy modality (e.g., including chemotherapy and radiotherapy) and close follow-up may be necessary for patients with low ASPP2 expression. Our study confirmed that high P53 expression was an independent predictor of short DFS, which was consistent with the results reported in literature [23, 41, 42]. Similar to ASPP2, patients with high P53 expression need more aggressive therapy modality and close follow-up to reduce the chance of relapse or metastasis. Although high ASPP1 expression was not associated with the prognosis of the whole cohort, it was associated with prolonged OS and DFS of patients with stage III disease. We hypothesize that ASPP1 mainly suppress tumor pregression in patients with advanced ESCC.

As their names “apoptosis-stimulating protein of P53 1 and 2” suggest, ASPP1 and ASPP2 were initially reported to function in a P53-dependent manner. Samuels-Lev et al. [11] found ASPP2 down-regulation in tumors with wild-type P53 but not in those with mutant P53. Iwabuchi et al. [43] suggested that ASPP1 and ASPP2 exerted their function of inhibiting tumorigenesis only in the presence of wild-type P53. However, later studies showed that ASPP2 played tumor-suppressing roles independent of P53, such as enhancing colorectal cancer cell apoptosis through autophagy inhibition [18], inhibiting cell migration through Src inactivation [14], and stimulating Ras-induced senescence through direct interactions with Ras-GTP [44]. Additionally, we did not find any correlation among ASPP1, ASPP2, and P53, indicating that they may all function independently. However, in this study, we only detected ASPP1 and ASPP2 expression using immunohistochemistry and the results need further experiments to verify and explore.

## Conclusions

ASPP1 may mainly function in advanced ESCCs rather than the gamut or early-stage ESCCs. ASPP2 may be an independent prognostic biomarker in ESCC, and patients with low ASPP2 expression may need more aggressive therapy modality and close follow-up. In the future, we will conduct in vivo and in vitro experiments to verify the immunohistochemistry results and further explore the biological mechanism of ASPP1’s and ASPP2’s functions in ESCC.

## Abbreviations

ESCC: esophageal squamous cell carcinoma
ASPP1 and ASPP2: apoptosis-stimulating protein of P53 1 and 2
The 5-year OS/DFS rate: the 5-year overall/disease-free survival rate
HR: hazard ratio
CI: confidence interval
EC: esophageal carcinoma
SH3: src-homology 3
PUMA: P53 up-regulated modulator of apoptosis
PIG3: P53-induced gene 3
NFKB1: nuclear factor of kappa light polypeptide gene enhancer in B-cells 1
SYSUCC: Sun Yat-sen University Cancer Center
PBS: phosphate buffer solution
IRS: immunoreactive score
NRF2: nuclear factor erythroid-2 related factor 2

## References

[1] Torre LA, Bray F, Siegel RL, Ferlay J, Lortet-Tieulent J, Jemal A (2015). Global cancer statistics, 2012. CA Cancer J Clin.

[2] Chen W, Zheng R, Zeng H, Zhang S (2015). The updated incidences and mortalities of major cancers in China, 2011. Chin J Cancer..

[3] Worni M, Martin J, Gloor B, Pietrobon R, D’Amico TA, Akushevich I (2012). Does surgery improve outcomes for esophageal squamous cell carcinoma? An analysis using the surveillance epidemiology and end results registry from 1998 to 2008. J Am Coll Surg.

[4] Dubecz A, Gall I, Solymosi N, Schweigert M, Peters JH, Feith M (2012). Temporal trends in long-term survival and cure rates in esophageal cancer: a SEER database analysis. J Thorac Oncol..

[5] Luo LL, Zhao L, Xi M, He LR, Shen JX, Li QQ (2015). Association of insulin-like growth factor-binding protein-3 with radiotherapy response and prognosis of esophageal squamous cell carcinoma. Chin J Cancer..

[6] Chen G, Wang Z, Liu XY, Liu FY (2007). Recurrence pattern of squamous cell carcinoma in the middle thoracic esophagus after modified Ivor-Lewis esophagectomy. World J Surg.

[7] Mariette C, Balon JM, Piessen G, Fabre S, Van Seuningen I, Triboulet JP (2003). Pattern of recurrence following complete resection of esophageal carcinoma and factors predictive of recurrent disease. Cancer.

[8] Nakagawa S, Kanda T, Kosugi S, Ohashi M, Suzuki T, Hatakeyama K (2004). Recurrence pattern of squamous cell carcinoma of the thoracic esophagus after extended radical esophagectomy with three-field lymphadenectomy. J Am Coll Surg.

[9] Lin SH, Chang JY (2010). Esophageal cancer: diagnosis and management. Chin J Cancer..

[10] Bellini MF, Cadamuro AC, Succi M, Proenca MA, Silva AE (2012). Alterations of the TP53 gene in gastric and esophageal carcinogenesis. J Biomed Biotechnol.

[11] Samuels-Lev Y, O’Connor DJ, Bergamaschi D, Trigiante G, Hsieh JK, Zhong S (2001). ASPP proteins specifically stimulate the apoptotic function of p53. Mol Cell.

[12] Slee EA, Lu X (2003). The ASPP family: deciding between life and death after DNA damage. Toxicol Lett.

[13] Sgroi DC, Teng S, Robinson G, LeVangie R, Hudson JR, Elkahloun AG (1999). In vivo gene expression profile analysis of human breast cancer progression. Cancer Res.

[14] Mak VC, Lee L, Siu MK, Wong OG, Lu X, Ngan HY (2013). Downregulation of ASPP2 in choriocarcinoma contributes to increased migratory potential through Src signaling pathway activation. Carcinogenesis.

[15] Cobleigh MA, Tabesh B, Bitterman P, Baker J, Cronin M, Liu ML (2005). Tumor gene expression and prognosis in breast cancer patients with 10 or more positive lymph nodes. Clin Cancer Res.

[16] Su M, Gu Y, Su S, Lu B (2014). Expression of ASPP gene family and its relationship with survival of patients with non-small cell lung cancer. Chin J Oncol..

[17] Lossos IS, Natkunam Y, Levy R, Lopez CD (2002). Apoptosis stimulating protein of p53 (ASPP2) expression differs in diffuse large B-cell and follicular center lymphoma: correlation with clinical outcome. Leuk Lymphoma.

[18] Shi Y, Han Y, Xie F, Wang A, Feng X, Li N (2015). ASPP2 enhances oxaliplatin (L-OHP)-induced colorectal cancer cell apoptosis in a p53-independent manner by inhibiting cell autophagy. J Cell Mol Med.

[19] Benyamini H, Leonov H, Rotem S, Katz C, Arkin IT, Friedler A (2009). A model for the interaction between NF-kappa-B and ASPP2 suggests an I-kappa-B-like binding mechanism. Proteins..

[20] Wang Y, Godin-Heymann N, Dan Wang X, Bergamaschi D, Llanos S, Lu X (2013). ASPP1 and ASPP2 bind active RAS, potentiate RAS signalling and enhance p53 activity in cancer cells. Cell Death Differ.

[21] Royer C, Koch S, Qin X, Zak J, Buti L, Dudziec E (2014). ASPP2 links the apical lateral polarity complex to the regulation of YAP activity in epithelial cells. PLoS ONE.

[22] Cong W, Hirose T, Harita Y, Yamashita A, Mizuno K, Hirano H (2010). ASPP2 regulates epithelial cell polarity through the PAR complex. Curr Biol CB..

[23] Yao W, Qin X, Qi B, Lu J, Guo L, Liu F (2014). Association of p53 expression with prognosis in patients with esophageal squamous cell carcinoma. Int J Clin Exp Pathol.

[24] Li S, Shi G, Yuan H, Zhou T, Zhang Q, Zhu H (2012). Abnormal expression pattern of the ASPP family of proteins in human non-small cell lung cancer and regulatory functions on apoptosis through p53 by iASPP. Oncol Rep.

[25] Tordella L, Koch S, Salter V, Pagotto A, Doondeea JB, Feller SM (2013). ASPP2 suppresses squamous cell carcinoma via RelA/p65-mediated repression of p63. Proc Natl Acad Sci USA.

[26] Wang X (2011). p53 regulation: teamwork between RING domains of Mdm2 and MdmX. Cell Cycle (Georgetown, Tex)..

[27] de Moraes E, Dar NA, de Moura Gallo CV, Hainaut P (2007). Cross-talks between cyclooxygenase-2 and tumor suppressor protein p53: balancing life and death during inflammatory stress and carcinogenesis. Int J Cancer J Int du Cancer..

[28] Soussi T, Legros Y, Lubin R, Ory K, Schlichtholz B (1994). Multifactorial analysis of p53 alteration in human cancer: a review. Int J Cancer J Int du Cancer..

[29] Levine AJ, Momand J, Finlay CA (1991). The p53 tumour suppressor gene. Nature.

[30] Starzynska T, Bromley M, Ghosh A, Stern PL (1992). Prognostic significance of p53 overexpression in gastric and colorectal carcinoma. Br J Cancer.

[31] Kitagishi Y, Kobayashi M, Matsuda S (2012). Protection against cancer with medicinal herbs via activation of tumor suppressor. J Oncol.

[32] Goh AM, Coffill CR, Lane DP (2011). The role of mutant p53 in human cancer. J Pathol.

[33] Huang K, Chen L, Zhang J, Wu Z, Lan L, Wang L (2014). Elevated p53 expression levels correlate with tumor progression and poor prognosis in patients exhibiting esophageal squamous cell carcinoma. Oncol Lett..

[34] Hinds PW, Finlay CA, Quartin RS, Baker SJ, Fearon ER, Vogelstein B (1990). Mutant p53 DNA clones from human colon carcinomas cooperate with ras in transforming primary rat cells: a comparison of the “hot spot” mutant phenotypes. Cell Growth Diff: Mol Biol J Am Assoc Cancer Res..

[35] Iggo R, Gatter K, Bartek J, Lane D, Harris AL (1990). Increased expression of mutant forms of p53 oncogene in primary lung cancer. Lancet.

[36] Cho HY, Jedlicka AE, Reddy SP, Kensler TW, Yamamoto M, Zhang LY (2002). Role of NRF2 in protection against hyperoxic lung injury in mice. Am J Respir Cell Mol Biol.

[37] Thai P, Statt S, Chen CH, Liang E, Campbell C, Wu R (2013). Characterization of a novel long noncoding RNA, SCAL1, induced by cigarette smoke and elevated in lung cancer cell lines. Am J Respir Cell Mol Biol.

[38] Zhang L, Li J (2016). The relevance of Nrf2 pathway and autophagy in pancreatic cancer cells upon stimulation of reactive oxygen species. Oxid Med Cell Longev.

[39] Hu R, Saw CL, Yu R, Kong AN (2010). Regulation of NF-E2-related factor 2 signaling for cancer chemoprevention: antioxidant coupled with antiinflammatory. Antioxid Redox Signal.

[40] Zhang C, Shu L, Kong AT (2015). MicroRNAs: new players in cancer prevention targeting Nrf2, oxidative stress and inflammatory pathways. Curr Pharmacol Rep.

[41] Xiang JF, Wang WQ, Liu L, Xu HX, Wu CT, Yang JX (2016). Mutant p53 determines pancreatic cancer poor prognosis to pancreatectomy through upregulation of cavin-1 in patients with preoperative serum CA19-9 >/= 1000 U/mL. Sci Rep.

[42] Deben C, Deschoolmeester V, Lardon F, Rolfo C, Pauwels P (2015). TP53 and MDM2 genetic alterations in non-small cell lung cancer: evaluating their prognostic and predictive value. Crit Rev Oncol/hematol..

[43] Iwabuchi K, Li B, Massa HF, Trask BJ, Date T, Fields S (1998). Stimulation of p53-mediated transcriptional activation by the p53-binding proteins, 53BP1 and 53BP2. J Biol Chem.

[44] Wang Z, Liu Y, Takahashi M, Van Hook K, Kampa-Schittenhelm KM, Sheppard BC (2013). N terminus of ASPP2 binds to Ras and enhances Ras/Raf/MEK/ERK activation to promote oncogene-induced senescence. Proc Natl Acad Sci USA.

